# PREVENTION-ACHD: PRospEctiVE study on implaNTable cardioverter-defibrillator therapy and suddeN cardiac death in Adults with Congenital Heart Disease; Rationale and Design

**DOI:** 10.1007/s12471-019-1297-3

**Published:** 2019-07-03

**Authors:** J. T. Vehmeijer, Z. Koyak, A. H. Zwinderman, L. Harris, R. Peinado, E. N. Oechslin, C. K. Silversides, B. J. Bouma, W. Budts, I. C. van Gelder, J. M. Oliver, B. J. M. Mulder, J. R. de Groot

**Affiliations:** 1 Heart Center, Department of Cardiology, Academic Medical Center—University of Amsterdam, Amsterdam, The Netherlands; 2 Department of Clinical Epidemiology and Biostatistics, Academic Medical Center—University of Amsterdam, Amsterdam, The Netherlands; 3 Division of Cardiology, Peter Munk Cardiac Center, Toronto Congenital Cardiac Center of Adults, University of Toronto, Toronto, Canada; 4 Department of Cardiology, La Paz University Hospital, Autonomous University of Madrid, Madrid, Spain; 5 Department of Cardiology, Universitair Ziekenhuis Leuven, Department of Cardiovascular Sciences, Katholieke Universiteit Leuven, Leuven, Belgium; 6 Department of Cardiology, University of Groningen, University Medical Center Groningen, Groningen, The Netherlands; 7 Department of Cardiology, Gregorio Marañon University Hospital and CIBERCV, Madrid, Spain; 8 Netherlands Heart Institute, Utrecht, The Netherlands

**Keywords:** Risk score, Risk stratification, Ventricular tachycardia, Ventricular fibrillation, Primary prevention

## Abstract

**Background:**

Many adult congenital heart disease (ACHD) patients are at risk of sudden cardiac death (SCD). An implantable cardioverter-defibrillator (ICD) may prevent SCD, but the evidence for primary prevention indications is still unsatisfactory.

**Study Design:**

PREVENTION-ACHD is a prospective study with which we aim to prospectively validate a new risk score model for primary prevention of SCD in ACHD patients, as well as the currently existing guideline recommendations. Patients are screened using a novel risk score to predict SCD as well as current ICD indications according to an international Consensus Statement. Patients are followed up for two years. The primary endpoint is the occurrence of SCD and sustained ventricular arrhythmias. The Study was registered at ClinicalTrials.gov (NCT03957824).

**Conclusion:**

PREVENTION-ACHD is the first prospective study on SCD in ACHD patients. In the light of a growing and aging population of patients with more severe congenital heart defects, more robust clinical evidence on primary prevention of SCD is urgently needed.

**Electronic supplementary material:**

The online version of this article (10.1007/s12471-019-1297-3) contains supplementary material, which is available to authorized users.

## What’s new?

- A novel risk score model presented here aims to accurately predict SCD in several high-risk congenital heart defects.
- This risk score model is based on seven risk factors, identified from the largest study on SCD in ACHD patients to date.
- The risk score model was internally validated, as well as externally validated in an independent cohort.
- PREVENTION-ACHD aims to validate the risk score model in a prospective setting.

## Introduction

Adult congenital heart disease (ACHD) patients face the risk of a myriad of complications late after surgical repair. Of these, sudden cardiac death (SCD) is perhaps the most devastating; it accounts for up to 25% of all deaths in this young population [1–4]. As a result of a higher birth prevalence of children with congenital heart defects and improved screening, surgical and medical techniques, the population of ACHD patients is growing rapidly [5, 6]. Moreover, the risk of SCD may rise with longer post-operative follow-up, as the number of risk factors accumulate, e.g. slowly worsening ejection fraction and heart failure, as well as atrial arrhythmias. Considering these aspects, the overall incidence of SCD in ACHD patients can be expected to increase, and its prevention may prove to be one of the next big challenges in the field of congenital heart disease.

The implantable cardioverter-defibrillator (ICD) was developed to prevent SCD resulting from ventricular tachyarrhythmias, which are responsible for approximately 80% of all SCDs in ACHD patients [1]. It is a well-established therapy in patients with ischaemic and non-ischaemic cardiomyopathy, for whom ICD implantation for primary prevention is supported by clearly defined guideline recommendations [7–9]. However, extrapolation of guideline recommendations for patients with acquired heart disease may not be optimal for ACHD patients. Nevertheless, guidelines for patients with acquired heart disease have generally been used for risk stratification for SCD in ACHD patients, because of a lack of superior alternatives.

An assessment of the risk of SCD specifically for ACHD patients is vital; the majority of ACHD patients have a low risk of SCD, but some patients, particularly those with more severe congenital lesions, are at high risk. The implantation of an ICD in ACHD patients does result in a high rate of appropriate ICD interventions, but data on the fate of those in whom an ICD is not implanted is lacking [10]. Moreover, ICD-related complications and inappropriate shocks are abundant in ACHD patients who receive an ICD [10]. This makes under- as well as over-implantation a tremendous problem, but physicians are currently not supported in the decision for or against ICD implantation by robust clinical evidence.

Currently there are three documents that list recommendations for primary prevention ICD implantation in ACHD patients: the PACES/HRS Expert Consensus Statement on the Recognition and Management of Arrhythmias in Adult Congenital Heart Disease, the 2015 European Society of Cardiology (ESC) Guidelines for the management of patients with ventricular arrhythmias and the prevention of sudden cardiac death, and the 2018 position paper of the European Heart Rhythm Association (EHRA), Association for European Paediatric and Congenital Cardiology (AEPC), and the ESC Working Group on Grown-up Congenital heart disease [9, 11, 12]. All three documents list essentially the same indications for ICD implantation, such as systemic left ventricular ejection fraction ≤35% accompanied by New York Heart Association (NYHA) class II or III heart failure symptoms. However, when these indications were applied to a cohort of ACHD patients who died of SCD compared to living matched controls, only a minority of SCD cases was correctly identified, and a poor discriminative ability between cases and controls was found [13]. Therefore, a more accurate risk stratification method for SCD is urgently needed to prevent more deaths from occurring in the rapidly growing population of adults with congenital heart disease.

In this study, we use a new risk stratification model, which is based on risk factors in ACHD patients who died of SCD, compared with living controls. The validation of prognostic risk prediction models is highly important [14, 15], particularly in this case, because the models assessed here predict preventable death in a group of young adults who are underrepresented in international guidelines. We aim to test a novel risk score that aims to accurately predict SCD in ACHD, as well as the current ICD indications in the international Consensus Statement on arrhythmias in ACHD and evaluate the risk of SCD at follow-up [11].

## Methods

### Risk score model

We developed a risk score model with which we aim to predict the annual risk of SCD or life-threatening arrhythmias in different types of congenital heart defects (CHD). This risk score model was based on a retrospective multicentre case-controlled study that evaluated risk factors for SCD in ACHD patients. In that study SCD cases were matched to living controls by age, gender, diagnosis, type of surgical intervention, date of surgical repair and treating medical centre [1].

**Fig. 1:**
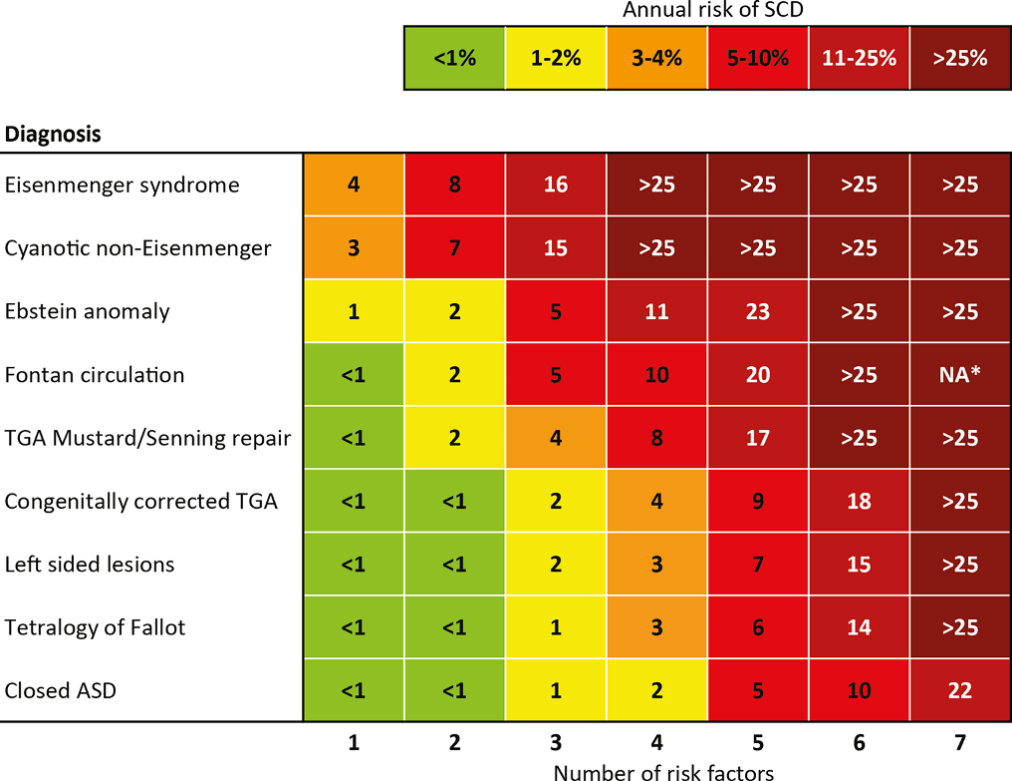
The risk score based on clinical risk factors represents the *annual *risk of sudden cardiac death. For each of the following seven risk factors one point is attributed to the model: *1* Coronary artery disease*, 2* Heart failure symptoms (New York Heart Association class II/III),* 3* Supraventricular tachycardia, *4* Impaired systemic ventricular function (ejection fraction <40%), *5* Impaired subpulmonary ventricular function (ejection fraction <40%), *6* QRS duration >120 ms*, 7* QT dispersion >70 ms* (ASD* atrial septal defect, *SCD* sudden cardiac death, *TGA* transposition of the great arteries, *Seven risk factors not possible for Fontan patients, as these patients do not have a subpulmonary ventricle)

To identify clinical variables associated with SCD, univariable and stepwise backward multivariable conditional logistic regression models were used. A detailed description of the identification of the risk factors is described elsewhere [1], and in the online supplementary material. The stability of the variable selection procedure was evaluated with 1000 bootstrap analyses. Variables selected at least 400 times were included in the final model. For all logistic regression models, odds ratios (OR) with 95% confidence intervals were calculated. To determine the risk of SCD, we developed a point-based risk scoring system. Points were attributed to each variable in the risk score model depending on the log odds ratio or B‑coefficient, which was derived from the multivariable analysis. The resulting risk score model consists of seven risk factors for SCD, for each of which one point is attributed to the model. This model was used to assess the absolute annual risk of SCD by multiplying the hazard ratio associated with one point in the risk score by the number of points and the *a-priori* risk of SCD for each CHD lesion (Fig. 1). The annual incidence of SCD per congenital defect was derived from the Concor registry [16]. The lesions with the highest risk of SCD are included in the final model.

The performance of the risk score model was evaluated using the area under the receiver operating characteristics (AUROC) curve. We internally validated the performance of our model by a second bootstrapping, which included the variable selection bootstrapping procedure. In addition to the internal validation, an independent prospective registry cohort from La Paz University Hospital, Madrid, Spain was used to externally validate the risk score model. From this cohort, which includes 3311 adults with CHD from December 1989 to December 2013, all SCD cases were included [17].

The clinical parameters included in the risk score are identifiable through routine follow-up examinations, such as the electrocardiogram and echocardiogram. Therefore it may especially be suitable for application during outpatient clinic visits. However, not all congenital heart defects are incorporated into this risk score, predominantly because patients with other defects have such a low *a-priori* risk of SCD that their risk score cannot be calculated, or the congenital defect is too uncommon to provide an accurate risk prediction.

In addition to validating this risk score in a prospective setting, the Consensus Statement indications will also be verified in this study for their accuracy in predicting SCD events and appropriate ICD interventions.

### Study design

**Fig. 2:**
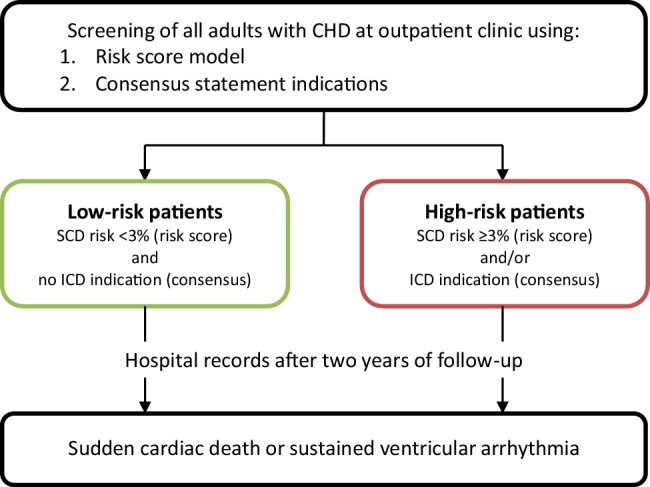
Flow chart of patient selection and follow-up (*CHD* congenital heart disease, *ICD* implantable cardioverter-defibrillator, *SCD* sudden cardiac death). (Consensus: PACES/HRS Expert Consensus Statement on the Recognition and Management of Arrhythmias in Adult Congenital Heart Disease [11])

This is a single centre, prospective, observational study. Patients with an outpatient clinic appointment at the Academic Medical Center in Amsterdam during an enrolment period of one year will be screened at baseline using the risk score and the ICD indications according to the Consensus Statement. A flow chart of the study design is presented in Fig. 2.

### Assessment of risk

Patients are deemed to be at high risk for SCD when they have at minimum a 3% annual risk of SCD according to the risk score model. ICD implantation may be considered in these patients. The control group will consist of ACHD patients with a low risk of SCD (<3% annually) according to the risk score.

The primary prevention indications from the aforementioned Consensus Statement are listed in Tab. 1.

**Table 1:** Primary prevention ICD indications according to the PACES/HRS Expert Consensus Statement on arrhythmia in ACHD [11]

*Class I*
ICD therapy is indicated in adults with CHD and a systemic left ventricular ejection fraction ≤35%, biventricular physiology, and New York Heart Association (NYHA) class II or III symptoms (Level of evidence: B)
*Class IIa*
ICD therapy is reasonable in selected adults with tetralogy of Fallot and multiple risk factors for sudden cardiac death, such as left ventricular systolic or diastolic dysfunction, nonsustained ventricular tachycardia, QRS duration ≥180 ms, extensive right ventricular scarring, or inducible sustained ventricular tachycardia at electrophysiology study (Level of evidence: B)
*Class IIb*
1. ICD therapy may be reasonable in adults with a single or systemic right ventricular ejection fraction <35%, particularly in the presence of additional risk factors such as complex ventricular arrhythmias, unexplained syncope, NYHA functional class II or III symptoms, QRS duration ≥140 ms, or severe systemic AV valve regurgitation (Level of evidence: C)
2. ICD therapy may be considered in adults with CHD and a systemic ventricular ejection fraction <35% in the absence of overt symptoms (NYHA class I) or other known risk factors (Level of evidence of: C)
3. ICD therapy may be considered in adults with CHD and syncope of unknown origin with haemodynamically significant sustained ventricular tachycardia or fibrillation inducible at electrophysiologic study (Level of evidence: B)
4. ICD therapy may be considered for nonhospitalised adults with CHD awaiting heart transplantation (Level of evidence: C)
5. ICD therapy may be considered for adults with syncope and moderate or complex CHD in whom there is a high clinical suspicion of ventricular arrhythmia and in whom thorough invasive and noninvasive investigations have failed to define a cause (Level of evidence: C)

*ICD* implantable cardioverter-defibrillator, *CHD* congenital heart disease, *ACHD* adult congenital heart disease

### Patient selection

All patients included in this registry are adults (≥18 years of age) who have been diagnosed with a structural congenital heart defect.

Exclusion criteria are the following:

- secondary prevention ICD indication, i.e. spontaneous sustained ventricular tachycardia or fibrillation or survived cardiac arrest warranting ICD implantation
- myocardial infarction in the previous three months
- high risk status or ICD indication according to the Consensus Statement due to a transient cause, e.g. tachycardiomyopathy or operable valvular dysfunction
- completion of follow-up is not possible
- a contra-indication for ICD implantation (including NYHA IV heart failure)

Patients with congenital defects not represented in the risk score will be excluded from the analysis comparing patients with high and low risk scores, but may still be screened by applying the Consensus Statement indications.

### Treatment of patients

As this is an observational study, follow-up examinations and regular treatment of patients will be performed by the patient’s own treating physician, without intervention by the investigators. The decision on whether to implant an ICD is at the treating physician’s discretion.

### Follow-up

The initial follow-up period will be two years. After these two years the endpoint of SCD or appropriate ICD interventions in patients with an ICD will be examined. Hospital records will be inspected for SCD events, sustained ventricular arrhythmia and appropriate ICD interventions.

### Ethics approval

We obtained a waiver from the ethics committee at the Academic Medical Center—University of Amsterdam, as this observational study does not require approval from the ethics committee. The study was registered at ClinicalTrials.gov (NCT03957824).

### Endpoints and power calculation

The study is powered for the composite endpoint of SCD or sustained ventricular arrhythmia at two years of follow-up. Sustained ventricular arrhythmia is defined according to international standards as any ventricular fibrillation or ventricular tachycardia lasting longer than 30 seconds. In patients with an ICD, device programming will contain algorithms to prevent unnecessary ICD therapy for self-terminating ventricular arrhythmias [18].

The ratio of high-risk versus low-risk patients is estimated to be 1:10. We estimate that the two-year risk of SCD or sustained ventricular arrhythmia for low-risk patients is 0.4% and for high-risk patients is 6%. Considering an attrition of 10%, we calculated that with 60 high-risk patients and 600 low-risk patients a high degree of confidence (>80% power) can be provided to validate the risk score.

Secondary endpoints, in ACHD patients with an ICD, are the rates of ICD-related complications and inappropriate shocks.

## Discussion

The prevention of SCD is a vital part of the care for ACHD patients, but major difficulties in the indication assessment for ICD implantation are yet to be overcome. Current risk prediction methods, including the current guidelines on ICD implantation in ACHD patients have shown to be of limited predictive value [13].

Considering this, a novel risk score designed to predict SCD in ACHD patients was developed, which was internally validated using bootstrapping, and externally validated in an independent registry cohort. With PREVENTION-ACHD, we aim to validate this risk score in a prospective study. In addition, the ICD indications listed in the Expert Consensus Statement on arrhythmias in ACHD patients will be assessed.

### Risk score model

By means of the presented risk score model the patient’s individual risk for SCD can be assessed. The risk score model concurs with risk factors previously reported on specific cardiac defects and adds quantitative data for other CHD lesions. Most studies seeking risk factors for SCD in CHD have involved patients with surgically repaired tetralogy of Fallot (ToF) and Mustard or Senning repair for transposition of the great arteries (TGA). However, SCD also frequently occurs in other CHD such as Eisenmenger syndrome, left-sided lesions and septal defects, which are either less prevalent or less well investigated. This risk score model includes many types of cardiac lesions and may be applied to a broad spectrum of patients.

### Consensus Statement

The *PACES/HRS Expert Consensus Statement on the Recognition and Management of Arrhythmias in Adult Congenital Heart Disease *listed ICD recommendations for ACHD patients for the first time [11]. In a retrospective analysis, the ICD recommendations in the Consensus Statement failed to identify 60% of SCD victims, ICD implantation was recommended in 17% of living controls, and the overall discriminative ability was poor [13]. However, the Consensus Statement ICD recommendations have not yet been tested in a prospective setting. PREVENTION-ACHD may therefore provide more accurate results regarding the discriminative ability of the ICD recommendations.

### ICD implantation

Patients are classified into low-risk and high-risk groups using the risk score to provide more distinctly defined ICD indications and to reduce the number of patients needed. We consider a ≥3% annual risk of SCD to be high, in part because of the young age of ACHD patients who are at risk of SCD and the cumulative rates that may be far higher than in the much older population with acquired heart disease. Moreover, according to current ESC guidelines, patients with hypertrophic cardiomyopathy are considered candidates for primary prevention ICD implantation when they have a 5-year SCD risk of 6% (class IIa). Among other reasons, the high rate of complications associated with ICD implantation in ACHD patients made us reluctant to lower the cut-off rate for high-risk patients, who may be considered for ICD implantation, to similar numbers.

### Limitations

Although the risk score model is derived from the largest cohort of ACHD patients who were the victim of SCD, the number of cases providing data is still limited [1]. The study was retrospective in nature, and thus the inherent limitations of such a study design apply. However, it should be noted that to date, no prospective studies on SCD in ACHD patients are available.

## Conclusion

Considering the expected rising incidence of SCD and high complication rate associated with ICD implantation in ACHD patients, it is of paramount importance to gather more robust evidence on the risk stratification for SCD and the indication for ICDs. PREVENTION-ACHD is the first prospective study on SCD in ACHD patients. A novel risk score predicting the risk of SCD in ACHD patients and the ICD indications listed in the 2014 Consensus Statement on arrhythmias in adult congenital heart disease, are both aimed to be validated with this study.

## Caption Electronic Supplementary Material

Supplemental material to PREVENTION-ACHD: rationale and design contains a detailed description of the development of the PREVENTION-ACHD risk score, and its internal and external (retrospective) validation.
