# Supplementary material for: PREVENTION-ACHD: PRospEctiVE study on implaNTable cardioverter-defibrillator therapy and suddeN cardiac death in Adults with Congenital Heart Disease; Rationale and Design
Source: Neth Heart J. 2019 Jul 3;27(10):474–9. doi: 10.1007/s12471-019-1297-3 (PMC6773785; doi:10.1007/s12471-019-1297-3)
Supplement: Supplementary file 1 — Supplemental material to PREVENTION-ACHD: rationale and design contains a detailed description of the development of the PREVENTION-ACHD risk score, and its internal and external (retrospective) validation. [file 12471_2019_1297_MOESM1_ESM.docx]

Supplement to:

PREVENTION-ACHD: PRospEctiVE study on implaNTable cardioverter-defibrillator therapy and suddeN cardiac death in Adults with Congenital Heart Disease

# Methods of model development

## Risk factors for SCD in CHD

We developed a risk score model to predict the annual risk of SCD or life threatening arrhythmias in different types of CHD. This risk score model was based on the original patient data of a retrospective multicentre case-controlled study that evaluated risk factors for SCD in adults with CHD. In that study for each SCD case multiple living control patients (1 to 4 controls per case, depending on availability) were matched to:

1. age
2. gender
3. congenital heart defect diagnosis
4. type of surgical intervention
5. date of surgical repair
6. treating medical centre.^1^

The current model was used to assess the absolute annual risk of SCD for patients with various congenital heart conditions.

## Annual incidence of SCD

The annual incidence of SCD in adults with CHD was explicitly reported in only a limited number of studies (Table S5). In this study we derived the incidence of SCD from the Concor registry.^12^

## Definitions

Proven or documented arrhythmic death was defined as instantaneous death with documented ventricular fibrillation or ventricular tachycardia. Presumed arrhythmic death included instantaneous death or circumstances compatible with SCD, without severe disease that would lead to death soon and in the absence of a non-arrhythmic cause of death at autopsy or abrupt loss of consciousness and disappearance of pulse but no further data. Cases were defined as adult CHD patients with SCD and controls were alive adult CHD patients without SCD. Supraventricular tachycardias (SVT) included documented atrial fibrillation, atrial flutter, atrial tachycardia and unspecified SVT. Heart failure was defined as a syndrome with typical symptoms (e.g. breathlessness, ankle swelling, and fatigue) and signs (e.g. elevated jugular venous pressure, pulmonary crackles, and displaced apex beat) resulting from an abnormality of cardiac structure or function. Documented coronary artery disease included prior myocardial infarction, history of coronary revascularisation, luminal stenosis ≥50% on coronary angiography or ischemia on myocardial perfusion scan. QRS duration and QT dispersion were analysed manually from standard (25mm/s and 1mV/cm) 12-lead ECGs. QRS duration and QT dispersion were dichotomised, optimal cut-off points were determined based on the best balance of sensitivity and specificity (≥80%) resulting in QRS duration ≥120ms and QT dispersion ≥70ms. We chose to dichotomise QRS duration and QT dispersion because of the ease of use of dichotomous variables in practice compared to models with continuous variables. The prognostic value was only slightly lowered by this choice. Ventricular function was assessed by two-dimensional echocardiography and classified as normal (ejection fraction (EF) ≥50%), mildly (EF 40-49%), moderately (EF 30-39%) and severely (EF <30%) impaired. Since the aorta does not always arise from the morphological left ventricle, the ventricles were classified as systemic and subpulmonary throughout the manuscript instead of left and right ventricle.

## General statistical analysis

Data analyses were performed with SPSS software for Windows (20.0 for Windows; SPSS Inc., Chicago. Illinois, US). For all analyses two-tailed probability values <0.05 were considered statistically significant. Descriptive statistics for nominal data were expressed in absolute numbers and percentages. After confirming normality, mean values and standard deviations were calculated for normally distributed continuous variables. When comparing frequencies and means, the χ²-test and Student’s t-test were used, respectively.

## Model development

To identify clinical variables associated with SCD univariable and stepwise backward multivariable conditional logistic regression models were used. The stability of the variable selection procedure was evaluated using 1000 bootstrap analysis and variables selected at least 400 times were included in the final model. For all logistic regression models, odds ratios (OR) with 95% confidence intervals were calculated. To determine the risk of SCD, we developed a point-based risk scoring system. Points were attributed to each variable in the risk score model depending on the log odds ratio or B-coefficient which was derived from multivariable analysis. In this study, the clinical variables associated with SCD in multivariable analysis were slightly different compared to the original cohort.^1^ For the risk score model we used dichotomised QRS duration and QT dispersion instead of continuous variables. This resulted in two additional risk factors for SCD in multivariable analysis; coronary artery disease (CAD) and heart failure symptoms.

## Model Presentation

The scoring system was used to determine the estimated probability (*P*) of SCD. *P* depends on the score *Prognostic Index (PI)* according to the formula

$$P(SCD|x_{1},...,x_{k}) = \frac{e^{b_{0}+b_{1}x_{1}+...+b_{k}x_{k}}}{1+e^{b_{0}+b_{1}x_{1}+...+b_{k}x_{k}}}$$

in which *PI* = *b_1_x_1_ ...+ b_k_x_k_* is a weighted sum of the values *x*_1_, . . ., *x*_k_ of *k* risk factors or predictors, each coded as 1 if present and 0 if absent in a patient, with *b*_1_, . . . , *b*_k_ as the estimated B-coefficients, each describing the log-odds-ratio associated with the corresponding factor. *b_0_* is the intercept term describing the baseline log-odds of SCD, that is, *P= (1+exp ( - b_0_ ))^-1^* is the baseline risk of SCD in a patient with no risk factors. Because the current data were sampled in a case-control study, we did not use *b_0_* in calculation if individual risk score.

## Internal assessment of the risk score model

The performance of the risk score models was evaluated using the area under the receiver operating characteristic (ROC) curve, known as the C-index. Models with an area under the ROC curve of ≥0.7 are considered to have substantial predictive value. A well-known problem of predictive multivariable models is that their performance is frequently overestimated because they are evaluated on the sample also used for the construction of the prediction model. This phenomenon, known as ‘optimism’, is important for appropriate validation of multivariable models. We, therefore, internally validated the performance of our model by a second bootstrap step which included the variable selection bootstrap procedure. The areas under the ROC curves (AUCs) were calculated and are estimates of how well patients with SCD will be discriminated from patients without SCD by the score (discriminating power). ROC curves were plotted and the AUCs of the simplified scores were compared with the AUCs achieved with the scores based on the estimated B-coefficients. Equally weighted factors means that each factor in the score derived from the logistic regression analysis, was given a coefficient of 1, leading to the following type of score: PI= X_1_ +….X_k_. Each factor contributes 1 to this score if present and 0 if absent in a patient. Hence, the number of risk factors present provides the individual value of this simplified score.

The determination of the estimated probability (P) of SCD is detailed in the supplementary appendix. We calculated 1- year SCD risk assuming exponential survival using baseline hazards according to the incidence of SCD among various cardiac defects as summarised in Table S5.

## External assessment of the risk score model

An independent prospective registry cohort (tertiary centre La Paz University Hospital, Madrid, Spain) was used to validate our risk score model for SCD. This cohort includes 3311 adults with CHD from December 1989 to December 2013.^2^ Patients in this cohort were 50.5% males, with a median age at entry of 22.5 years and median follow-up time 10.5 years (IQR: 4.4–18)]. During a cumulative follow-up time of 37,608 person-years, a total of 336 patients died (10%), with an annual death rate of 0.89%. From this cohort all SCD cases (*n* = 53) were identified and included according to the definition arrhythmic death and inclusion criteria as described above. For determining vital status demographic data were crossed with the Spain national death registry.

## Risk score

Seven clinical variables associated with SCD in CHD in multivariable analysis were identified: 1) documented CAD, 2) heart failure symptoms, 3) supraventricular tachycardias, 4) at least moderately impaired systemic ventricular function (SVF), 5) at least moderately impaired subpulmonary function (PVF), 6) QRS duration ≥120ms (ventricular paced rhythms excluded) and 7) QT dispersion ≥70ms. The prevalence of these variables has been summarised in Table S1. Of note, although statistically significant in multivariable analysis, heart failure medication use was not included in multivariable analysis as this variable is strongly influenced by the physician’s decision making.

Based on these seven clinical variables derived from multivariable analysis, three risk score models were constructed. The values of B-coefficients and points attributed to each characteristic in the three risk score models are summarised in Table S1. Risk score 1 was based on the raw logistic regression coefficients. Risk score 2 was based on the logistic regression coefficients with rounded to whole or half numbers. In risk score 3 each clinical variable was weighted equally and thus 1 point was attributed to each clinical variable. A score was calculated for each patient by adding up the points corresponding to his or her risk factors. The calibration of these risk 3 scores is illustrated in Fig. S1. The three scores showed excellent calibration.

ROC curves were constructed for the three risk score models (Fig. S2A), using the individual patient data of the study from which the risk factors were derived. The discriminating power of each score was assessed using the C-index. Table S2 shows the C-index for the three risk score models, which was 0.77 for risk score model 1, 0.77 for risk model 2 and 0.76 for risk score model 3. There was no essential difference in C-index between the three risk score models. Hence, all three risk score models had a good performance and simplification did not weaken the discriminative power of the risk score models.

## Performance of the risk score model

The validation cohort included 3334 adults with CHD of whom 49% was male. Median age at first examination was 22 years (range 18 to 39 years). Median follow-up duration was 11 years (range 4 to 18 years). For determining vital status demographic data were crossed with the Spain national death registry. Overall 336 patients had died or had heart transplantation at the end of follow-up (10%). The annual incidence of death was 0.9%. Sudden arrhythmic death according to the definitions outlined above occurred in 37 patients (1%). However, for the validation only 24 SCDs were included as only these patients had complete clinical, electrocardiographic and echocardiographic data which was required for this analysis. These 24 SCDs were matched to 24 controls according to, age, gender, diagnosis, type of surgical intervention and date of surgical repair. Age of SCDs and controls was 38±15 and 45±15 years, respectively (*P*=0.118) and 63% was male. The underlying cardiac defects according to severity were mild in 17%, moderate in 17% and severe in 66% of the SCD cases. The specific cardiac lesions included (congenitally) corrected Transposition of great arteries (21%), TOF (17%), Eisenmenger syndrome (17%), Cyanotic non-Eisenmenger syndrome (13%), Septal defects (8%), Left sided lesions (8%), Fontan circulation (8%) and other (8%). Corrective surgery was performed in majority of the patients (71%). The remaining patients, except for those with Eisenmenger syndrome, had undergone palliative surgery. Fifty-four percent of the SCDs was in NYHA class III or IV compared to 21% of controls (*P*=0.017). Heart failure drugs including diuretic and ace-inhibitors were used in 79% of SCDs versus 29% of controls (*P*=0.001). Thirteen percent of SCDs used class I, II or III anti-arrhythmic drugs versus 4% of controls. Five out 24 SCDs died despite an implantable cardioverter-defibrillator for secondary prevention of SCD. Fig. S3 displays the prevalence of the risk factors of SCD in the patients studied.

The validation cohort did not differ from the cohort from which the risk factors were derived with regards to age (*P*=0.123) or gender (*P*=0.868). Also, the prevalence of different types of CHD was similar. Fig. S2B shows the ROC curves for the three risk score models of the independent validation cohort. The C-index was 0.72 (CI 0.57-0.87) for risk score model 1, 2 and 3.

## Calculation of absolute risk for SCD

There was no difference in performance of the three risk score models. Therefore, the simplest risk score model 3 in which 1 point is attributed to each variable was used to calculate the absolute annual risk of SCD for patients with various congenital heart conditions (Fig. S4). The calculation of the absolute risk of SCD was based on the number of risk factors present in one single patient and the a priori risk of SCD among various cardiac defects according to the Concor registry (Table S5).^12^ In the cohort from which the risk factors were derived, the number of risk factors was 0 in 21%, 1 in 32%, 2 in 21%, 3 in 13%, 4 in 9%, 5 in 3%, 6 in 1% and 7 in 0% of the study population. Hence, the patients with a high number of risk factors were underrepresented. Patients with severe CHD such as cyanotic defects had the highest risk of SCD. In these patients, for example, the presence of only two risk factors in a single patient was enough to confer an annual risk of SCD ≥4%. In patients with mild to moderate cardiac defects such as atrial septal defects and TOF at least 4 risk factors were required to reach the same risk of SCD.

## Applicability in specific conditions

The absolute chance of SCD relies on a priori risk and number of risk factors in a single patient. We recalculated the absolute risk of SCD by using published a priori risks (Table S5) for the following conditions: TOF, TGA and left sided lesions.^2,13^ There was no difference in cut-off values (displayed in Fig. S4) of calculated absolute risk of SCD for these diagnoses among those studies, supporting the applicability of the risk score among various patients with CHD. The predicted risk of SCD in patients with left sided lesions in the risk score model is higher than that of ToF patients. ToF is one of the most well-known high-risk lesions for ventricular tachycardia and SCD; this also means that ToF patients are the most common recipients of ICDs, are more often protected against SCD and, therefore, may have been underrepresented in the study cohort from which the model is derived.12 In addition, the majority of the left sided lesions in the original cohort were congenital aortic stenosis with a mechanical aortic valve. Patients with aortic stenosis in acquired heart disease are at risk of SCD with an annual incidence of SCD of 1% in asymptomatic patients to 34% in symptomatic patients.20 This may also be the case in ACHD patients.

A randomised controlled trial, in which one group receives an ICD and the other group does not, would provide a more precise assessment of the validity of the risk score model and the Consensus Statement ICD recommendations. However, the small number of patients and inadequate funding for these projects in ACHD patients preclude such a study. It is unlikely that a randomised trial on ICD implantation in ACHD patients will ever be achieved. Therefore, this study may provide the best possible evidence for ICD implantation in ACHD patients.

## Literature Search

Data from all previously published studies describing predictors of SCD, ventricular arrhythmias and appropriate ICD therapy were assessed, summarised and included in this analysis (Table S3). The available literature mainly consisted of retrospective studies. Although the risk factors seem different among these studies, they actually largely overlap. Evidently, there are lesion specific risk factors and not all predictors may be truly independent. For example, low ventricular function as corrective surgery may impact on QRS duration. The magnitude of the risk of these factors reported varied between the studies, probably due to the lesion specific characteristics and different (small) numbers of patients included. However, the main difference between the risk factors reported in different studies relates to their magnitude of risk with usually large confidence intervals and not to the predictor itself. Further, the prevalence of these risk factors within the study populations varied as well (Table S4). The annual incidence of SCD in adults with CHD is explicitly reported in only a limited number of studies (Table S5) and the reported annual incidence in patients with the same diagnosis varied. A consistent finding was that the more severe the cardiac defect the higher the annual risk of SCD.

# Tables

**Table S1** Clinical variables associated with SCD in multivariable analysis and point attributed to each variable in the different risk score models

|  | **Prevalence %** | **OR** | **95% CI** | **P-value** | **Points Attributed Model 1** | **Points Attributed Model 2** | **Points Attributed Model 3** |
| --- | --- | --- | --- | --- | --- | --- | --- |
| CAD | 3 | 3.48 | 0.88-13.80 | 0.077 | 3.48 | 3.5 | 1 |
| HF symptoms | 22 | 2.79 | 1.56-4.97 | 0.001 | 2.79 | 3.0 | 1 |
| SVT | 30 | 1.45 | 0.86-2.45 | 0.165 | 1.45 | 1.5 | 1 |
| QRS duration ≥120ms | 46 | 1.57 | 0.96-2.57 | 0.071 | 1.57 | 1.5 | 1 |
| QT dispersion ≥70ms | 24 | 3.56 | 2.07-6.09 | 0.000 | 3.56 | 3.5 | 1 |
| At least moderately impaired SVF | 21 | 2.17 | 1.21-3.89 | 0.009 | 2.17 | 2.0 | 1 |
| At least moderately impaired PVF | 11 | 3.24 | 1.49-7.06 | 0.003 | 3.24 | 3.0 | 1 |
| *OR* odds ratio, *CI* confidence interval, *SCD* sudden cardiac death *CAD* coronary artery disease, *HF* heart failure, *SVT* supraventricular tachycardia *SVF* systemic ventricular function, *PVF* subpulmonary ventricular function | | | | | | | |

**Table S2** Performance of the three risk score models

| **Risk Score Model** | **AUC** | **SE** | **Optimism Corrected AUC** | **Shrinkage Factor** |
| --- | --- | --- | --- | --- |
| 1 | 0.771 | 0.026 | 0.756 | 0.912 |
| 2 | 0.768 | 0.026 | 0.768 | 1.003 |
| 3 | 0.756 | 0.026 | 0.755 | 1.001 |
| *AUC* area under the receiver operating curve, *SE* standard error | | | | |

| **Table S3** Reported univariate and multivariable predictors of VT, SCD and appropriate ICD shocks in CHD | | | | | | | | | | | |
| --- | --- | --- | --- | --- | --- | --- | --- | --- | --- | --- | --- |
|  | **Koyak *et al.[1]*** | **Gallego *et al.[2]*** | **Schwerzmann *et al.[3]*** | **Khairy *et al.[4]*** | **Kammeraad *et al.[5]*** | **Ghai *et al.[6]*** | **Gatzoulis *et al.[7]*** | **Koyak *et al.* [8]** | **Khanna *et al.* [9]** | **Khairy *et al.* [10]** | **Khairy *et al.* [11]** |
|  | HR or OR (95% CI) | HR or OR (95% CI) | HR or OR (95% CI) | HR or OR (95% CI) | HR or OR (95% CI) | Prevalence (%) in Cases vs controls | HR or OR (95% CI) | HR or OR (95% CI) | HR or OR (95% CI) | HR or OR (95% CI) | HR or OR (95% CI) |
| **Study summary** |  |  |  |  |  |  |  |  |  |  |  |
| Patients (n) | 481 | 936 | 149 | 252 | 140 | 137 | 793 | 136 | 73 | 121 | 37 |
| Number of events (%) | 171 | 22 | 13 | 62 | 47 | 12 | 49 | 39 (29%) | 14 (19%) | 37 | 12 |
| **Study Design** | Cohort | Cohort | Cohort | Cohort | Case-control | Case-control | Cohort | Cohort | Cohort | Cohort | Cohort |
| Study population | General CHD | General CHD | TGA | TOF | TGA | TOF | TOF | General CHD | General CHD | TOF | TGA |
| Follow-up (yrs) | na |  | 9±6 | 6.5±4.5 | na | na | 21±9 | 4.6 (0.01-13.9) | 2.2±2.8 | 3.7(1.5 -5.6) | 3.6 (1.5-5.5) |
| Main outcome | SCD | SCD | SCD/VT | SCD/VT | SCD | SCD | SCD†/VT‡ | ICD therapy | ICD therapy | ICD therapy | ICD therapy |
| **Clinical** |  |  |  |  |  |  |  |  |  |  |  |
| HF symptoms |  |  |  |  | 4.4 (1.9-10.6)* |  |  |  |  |  |  |
| NYHA III/IV |  |  | 9.8 (3.0-31.6)* |  | 21.6 (2.8-166.8)* |  |  |  |  |  |  |
| Arrhythmic symptoms |  |  |  |  |  |  |  |  |  |  |  |
| Secondary prevention |  |  |  |  |  |  |  | 3.6 (1.3-9.5)** |  |  | 18.0 (1.2-216.0)** |
| Lack of beta blockers |  |  |  |  |  |  |  |  |  |  | 16.7 (1.3-185.2)** |
| CAD |  |  |  |  |  |  |  | 2.7 (1.0-7.2)** |  |  |  |
| Complex TGA |  |  | 4.9 (1.5-16.0)* |  |  |  |  |  |  |  |  |
| **Prior Arrhythmias** |  |  |  |  |  |  |  |  |  |  |  |
| Sustained VT |  |  |  |  |  | 42% vs 6% |  |  |  |  |  |
| NSVT |  |  |  |  |  |  |  | 9.1 (2.8-29.1)** |  | 3.7 (1.2-11.3)** |  |
| SVT | 3.5 (1.5-7.9)** |  |  |  | 4.9 (1.9-12.5)* |  |  |  |  |  |  |
| **Surgical** |  |  |  |  |  |  |  |  |  |  |  |
| Transannular patch |  |  |  |  |  |  | 11.7 (1.3-103.1)**† |  |  |  |  |
| Prior palliation |  |  |  |  |  |  | 3.1 (1.2-7.6)**‡ |  |  |  |  |
| **Haemodynamic** |  |  |  |  |  |  |  |  |  |  |  |
| SVEF ≤40% | 3.4 (1.1-10.4)** |  | 3.6 (1.1-11.2)* |  |  | 42% vs 9% |  |  |  |  |  |
| SVEF ≤35% |  | 29.9 (11-72)** |  |  |  |  |  |  |  |  |  |
| LVEDP ≥12mmHg |  |  |  |  |  |  |  |  |  | 4.9 (1.3-19.4)** |  |
| PVEF ≤40% | 3.4 (1.1-10.2)** |  |  |  |  |  |  |  |  |  |  |
| RVEDP ≥55mmHg |  |  |  |  |  |  |  |  | 6.1 (1.4-41.3)* |  |  |
| **Electrocardiographic** |  |  |  |  |  |  |  |  |  |  |  |
| QRS ≥140ms |  |  | 13.6 (2.9-63.4)* |  |  |  |  |  |  |  |  |
| QRS ≥180ms |  |  |  |  |  | 56% vs 13% | 2.3 (1.05-5.02)† 41.9 (14.7-119.4)**‡ |  |  |  |  |
| **Electrophysiology** |  |  |  |  |  |  |  |  |  |  |  |
| Inducible sustained VT |  |  |  | 4.7 (1.2-18.5)** |  |  |  |  |  |  |  |
| *VT* ventricular tachycardia, SCD sudden cardiac death, *ICD* implantable cardioverter-defibrillator, *CHD* congenital heart disease, *HR* hazard ratio, *OR* odds ratio, *CI* confidence interval, *HF* heart failure, *NYHA* New York Heart Association, *CAD* coronary artery disease, *TGA* transposition of great arteries, *TOF* tetralogy of Fallot, *SVT* supraventricular tachycardia, *NSVT* non-sustained ventricular tachycardia, *SVEF* systemic ventricular ejection fraction, PVEF subpulmonary ventricular ejection fraction, *LVEDP* left ventricular end-diastolic pressure, *RVEDP* right ventricular end-diastolic pressure  *univariate analysis **multivariable analysis | | | | | | | | | | | |
|  |  |  |  |  |  |  |  |  |  |  |  |

| **Table S4** Prevalence of predictors of SCD/VT or Appropriate ICD shocks among several studies | | | | | | | | | | | |
| --- | --- | --- | --- | --- | --- | --- | --- | --- | --- | --- | --- |
|  | **Koyak *et al.[1]*** | **Gallego *et al.[2]*** | **Schwerzmann *et al.[3]*** | **Khairy *et al.[4]*** | **Kammeraad *et al.[5]*** | **Ghai *et al.[6]*** | **Gatzoulis *et al.[7]*** | **Koyak *et al.* [8]** | **Khanna *et al.* [9]** | **Khairy *et al.* [10]** | **Khairy *et al.* [11]** |
| **Clinical** |  |  |  |  |  |  |  |  |  |  |  |
| HF symptoms | 22% |  |  |  |  | 58% |  |  |  |  |  |
| NYHA III/IV | 12% | 13% | 9% | 13% |  | 16% | 3% | 10% |  |  |  |
| Arrhythmic symptoms | 13% |  |  | 50% |  | 53% |  |  |  | 63% |  |
| Secondary Prevention |  |  |  |  |  |  |  | 50% | 36% | 31% | 30% |
| Lack of beta blockers |  |  | 82% |  |  | 81% |  | 65% |  |  | 43% |
| CAD | 2% |  |  |  |  | 4% |  | 10% |  |  |  |
| Complex TGA |  |  | 28% |  | 21% |  |  |  |  |  | 27% |
| **Prior Arrhythmias** |  |  |  |  |  |  |  |  |  |  |  |
| Spontaneous sustained VT | 2% |  | 5% | 17% |  | 9% | 4% | 27% | 28% |  |  |
| NSVT | 11% |  |  |  |  |  |  | 26% | 11% | 37% |  |
| SVT | 30% |  | 44% |  | 9% | 19% | 3% | 58% |  |  |  |
| **Surgical** |  |  |  |  |  |  |  |  |  |  |  |
| Transannular patch |  |  |  | 57% |  | 54% | 35% |  |  | 67% | 56% |
| Prior Palliation |  | 24% |  | 46% |  | 50% | 37% |  |  | 45% | 48% |
| **Haemodynamic** |  |  |  |  |  |  |  |  |  |  |  |
| SVEF ≤40% | 21% |  | 23% |  | 16% | 12% |  | 36% | 37% |  | 60% |
| PVEF ≤40% | 11% |  |  |  |  |  |  | 25% |  | 34% | 11% |
| SVEF ≤35% |  | 9% | 16% |  | 4% |  |  |  |  | 3% |  |
| LVEDP ≥12mmHg |  |  |  |  |  |  |  |  |  |  |  |
| RVEDP ≥55mmHg |  |  |  |  |  |  |  |  |  |  |  |
| **Electrocardiographic** |  |  |  |  |  |  |  |  |  |  |  |
| QRS ≥140ms | 18% |  | 17% |  |  |  |  |  |  |  |  |
| QRS ≥180ms |  |  |  | 19% |  | 15% | 11% | 21% |  | 28% | 30% |
| **Electrophysiology** |  |  |  |  |  |  |  |  |  |  |  |
| Inducible sustained VT |  |  |  |  |  |  |  | 73% | 19% | 41% | 41% |
| *SCD* sudden cardiac death, *VT* ventricular tachycardia, *ICD* implantable cardioverter-defibrillator, *HF* heart failure, *NYHA* New York Heart Association, *CAD* coronary artery disease, *TGA* transposition of the great arteries, *SVT* supraventricular tachycardia, *NSVT* nonsustained ventricular tachycardia, *SVEF* systemic ventricular ejection fraction, *PVEF* subpulmonary ventricular ejection fraction, *LVEDP* left ventricular end-diastolic pressure, *RVEDP* right ventricular end-diastolic pressure | | | | | | | | | | | |

| **Table S5** Incidence of SCD per 1000 patient years in CHD | | | |
| --- | --- | --- | --- |
|  | **Van der Velde *et al.[12]*** | **Silka *et al.[13]*** | **Gallego *et al.[2]*** |
| Overall | 2.7 | 0.9 | 2.6 |
| Repaired ToF | 1.3 | 1.5 | 4.7 |
| Closed ASD | 1.0 | 0.0 | 0.0 |
| TGA | 4.6 | 4.9 | 9.5 |
| *Mustard Procedure* | 3.7 | NA | NA |
| *Arterial Switch* | 5.4 | NA | NA |
| cc TGA | 1.8 | NA | 25 |
| Cyanotic | 16.6 | NA | NA |
| *Eisenmenger Syndrome* | 17.3 | NA | NA |
| *Cyanotic-Non-Eisenmenger* | 15.5 | NA | 5.4 |
| Fontan Circulation | 4.4 | NA | 2.8 |
| Left Sided Lesions | 1.4 | NA | NA |
| Ebstein's Anomaly | 5.1 | NA | NA |
| VSD | NA | 0.2 | 3.6 |
| CoA | NA | 1.3 | 2.1 |
| AVSD | NA | 0.9 | 1.8 |
| AS | NA | 5.4 | 0 |
| PS | NA | 0.3 | 0 |
| PDA | NA | 0.0 | NA |
| *SCD* sudden cardiac death, *ToF* tetralogy of Fallot, *ASD* atrial septal defect, *TGA* transposition of great arteries, *cc TGA* congenitally corrected transposition of great arteries, *VSD* ventricular septal defect, *CoA* coarctation of the aorta, *AVSD* atrioventricular septal defect, *AS* aortic stenosis, *PS* pulmonary stenosis, *PDA* patent arterial duct, *NA* not available | | | |


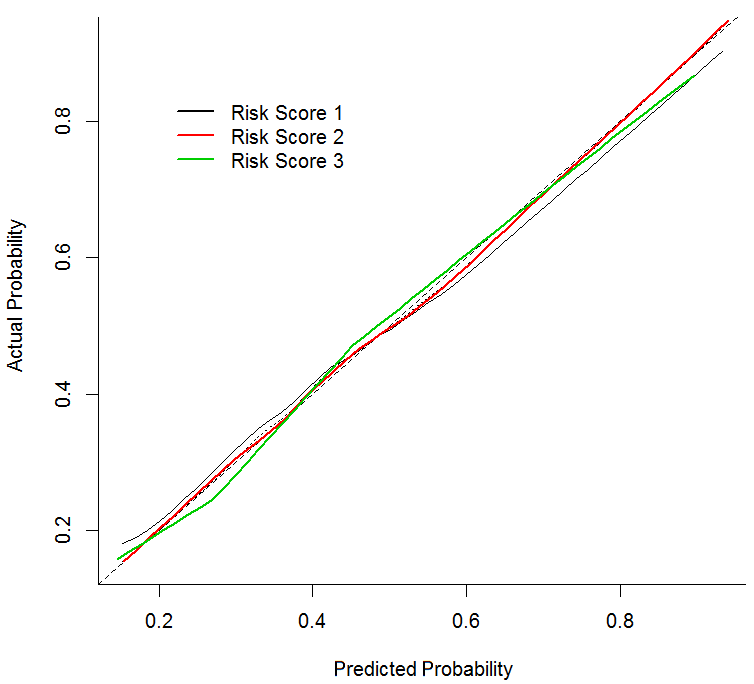


**Fig. S1** Calibration plots for risk score 1, 2 and 3 depicting predicted risk against observed risk of SCD in validation dataset

Dashed line (45°) from zero denotes ideal calibration line (slope=1, intercept=0).

**
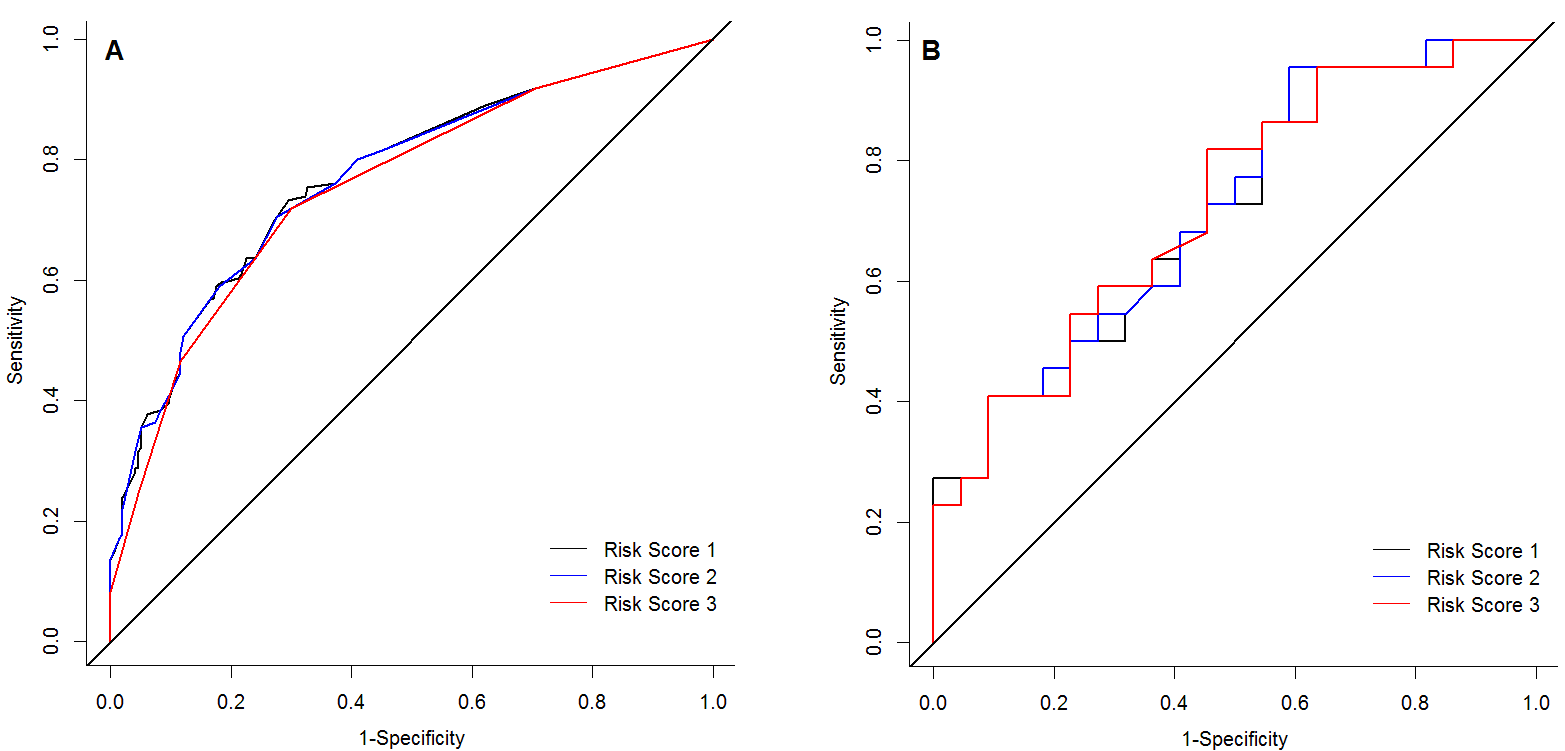
**

**Fig. S2** Receiver operating characteristic curves of the three risk scores in the internal validation cohort (A) and external validation cohort (B)


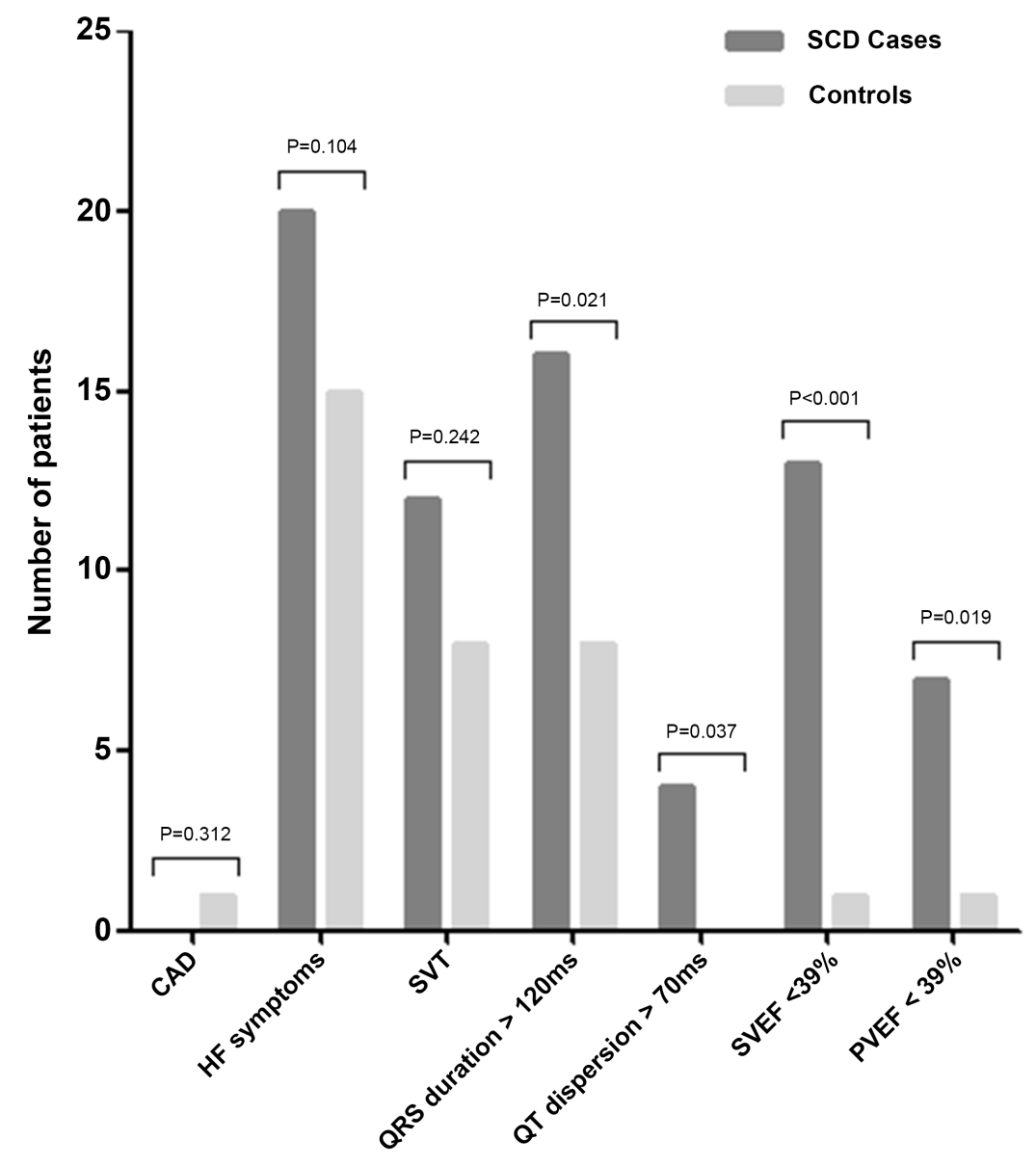


**Fig. S3** The prevalence of risk factors of the SCD cases versus controls in the external validation cohort

*HF* heart failure, *CAD* coronary artery disease, *SVT* supraventricular tachycardia, *SVEF* systemic ventricular ejection fraction, *PVEF* supraventricular tachycardia, *SVEF* systemic ventricular ejection fraction, *PVEF* subpulmonary ventricular ejection fraction

# References

[1] Koyak Z, Harris L, de Groot JR, Silversides CK, Oechslin EN, Bouma BJ, et al. Sudden Cardiac Death in Adult Congenital Heart Disease, Circulation. 2012) .

[2] Gallego P, Gonzalez AE, Sanchez-Recalde A, Peinado R, Polo L, Gomez-Rubin C, et al. Incidence and predictors of sudden cardiac arrest in adults with congenital heart defects repaired before adult life, Am J Cardiol. 110 (2012) 109-117.

[3] Schwerzmann M, Salehian O, Harris L, Siu SC, Williams WG, Webb GD, et al. Ventricular arrhythmias and sudden death in adults after a Mustard operation for transposition of the great arteries, Eur Heart J. 30 (2009) 1873-1879.

[4] Khairy P, Landzberg MJ, Gatzoulis MA, Lucron H, Lambert J, Marcon F, et al. Value of programmed ventricular stimulation after tetralogy of fallot repair: a multicenter study, Circulation. 109 (2004) 1994-2000.

[5] Kammeraad JA, van Deurzen CH, Sreeram N, Bink-Boelkens MT, Ottenkamp J, Helbing WA et al. Predictors of sudden cardiac death after Mustard or Senning repair for transposition of the great arteries, J Am Coll Cardiol. 44 (2004) 1095-1102.

[6] Ghai A, Silversides C, Harris L, Webb GD, Siu SC, Therrien J. Left ventricular dysfunction is a risk factor for sudden cardiac death in adults late after repair of tetralogy of Fallot, J Am Coll Cardiol. 40 (2002) 1675-1680.

[7] Gatzoulis MA, Balaji S, Webber SA, Siu SC, Hokanson JS, Poile C, et al. Risk factors for arrhythmia and sudden cardiac death late after repair of tetralogy of Fallot: a multicentre study, Lancet. 356 (2000) 975-981.

[8] Koyak Z, de Groot JR, Van Gelder IC, Bouma BJ, van Dessel PF, Budts W, et al. Implantable cardioverter defibrillator therapy in adults with congenital heart disease: who is at risk of shocks?, Circ Arrhythm Electrophysiol. 5 (2012) 101-110.

[9] Khanna AD, Warnes CA, Phillips SD, Lin G, Brady PA. Single-center experience with implantable cardioverter-defibrillators in adults with complex congenital heart disease, Am J Cardiol. 108 (2011) 729-734.

[10] Khairy P, Harris L, Landzberg MJ, Viswanathan S, Barlow A, Gatzoulis MA, et al. Implantable cardioverter-defibrillators in tetralogy of Fallot, Circulation. 117 (2008) 363-370.

[11] Khairy P, Harris L, Landzberg MJ, Fernandes SM, Barlow A, Mercier LA, et al. Sudden death and defibrillators in transposition of the great arteries with intra-atrial baffles: a multicenter study, Circ Arrhythm Electrophysiol. 1 (2008) 250-257.

[12] van der Velde ET, Vriend JW, Mannens MM, Uiterwaal CS, Brand R, Mulder BJ. CONCOR, an initiative towards a national registry and DNA-bank of patients with congenital heart disease in the Netherlands: rationale, design, and first results, Eur J Epidemiol. 20 (2005) 549-557.

[13] Silka MJ, Hardy BG, Menashe VD, Morris CD. A population-based prospective evaluation of risk of sudden cardiac death after operation for common congenital heart defects, J Am Coll Cardiol. 32 (1998) 245-251.
